# DiseaseMeth version 3.0: a major expansion and update of the human disease methylation database

**DOI:** 10.1093/nar/gkab1088

**Published:** 2021-11-18

**Authors:** Jie Xing, Ruiyang Zhai, Cong Wang, Honghao Liu, Jiaqi Zeng, Dianshuang Zhou, Mengyan Zhang, Liru Wang, Qiong Wu, Yue Gu, Yan Zhang

**Affiliations:** School of Life Science and Technology, Computational Biology Research Center, Harbin Institute of Technology, Harbin 150001, China; School of Life Science and Technology, Computational Biology Research Center, Harbin Institute of Technology, Harbin 150001, China; School of Life Science and Technology, Computational Biology Research Center, Harbin Institute of Technology, Harbin 150001, China; School of Life Science and Technology, Computational Biology Research Center, Harbin Institute of Technology, Harbin 150001, China; School of Life Science and Technology, Computational Biology Research Center, Harbin Institute of Technology, Harbin 150001, China; School of Life Science and Technology, Computational Biology Research Center, Harbin Institute of Technology, Harbin 150001, China; School of Life Science and Technology, Computational Biology Research Center, Harbin Institute of Technology, Harbin 150001, China; School of Life Science and Technology, Computational Biology Research Center, Harbin Institute of Technology, Harbin 150001, China; School of Life Science and Technology, Computational Biology Research Center, Harbin Institute of Technology, Harbin 150001, China; School of Life Science and Technology, Computational Biology Research Center, Harbin Institute of Technology, Harbin 150001, China; School of Life Science and Technology, Computational Biology Research Center, Harbin Institute of Technology, Harbin 150001, China; Guangzhou Institute of Respiratory health, State Key Laboratory of Respiratory Disease, Guangzhou Medical University, Guangzhou, 510120, China

## Abstract

DNA methylation has a growing potential for use as a biomarker because of its involvement in disease. DNA methylation data have also substantially grown in volume during the past 5 years. To facilitate access to these fragmented data, we proposed DiseaseMeth version 3.0 based on DiseaseMeth version 2.0, in which the number of diseases including increased from 88 to 162 and High-throughput profiles samples increased from 32 701 to 49 949. Experimentally confirmed associations added 448 pairs obtained by manual literature mining from 1472 papers in PubMed. The search, analyze and tools sections were updated to increase performance. In particular, the FunctionSearch now provides for the functional enrichment of genes from localized GO and KEGG annotation. We have also developed a unified analysis pipeline for identifying differentially DNA methylated genes (DMGs) from the original data stored in the database. 22 718 DMGs were found in 99 diseases. These DMGs offer application in disease evaluation using two self-developed online tools, Methylation Disease Correlation and Cancer Prognosis & Co-Methylation. All query results can be downloaded and can also be displayed through a box plot, heatmap or network module according to whichever search section is used. DiseaseMeth version 3.0 is freely available at http://diseasemeth.edbc.org/.

## INTRODUCTION

As an important epigenetic modification, DNA methylation is closely related to disease (1). DNA methylation is implicated in the repression of genes, and is associated with actively transcribed gene bodies (2). Global DNA hypomethylation and local hypermethylation of CpG regions are common in the carcinogenic process (3). DNA methylation can also be used to uncover cancer heterogeneity. In non-small cell lung cancer (NSCLC) *KLK10* was found to act as a functional tumor suppressor gene, and epigenetic inactivation of *KLK10* is a common event contributing to NSCLC pathogenesis and, therefore, may be used as a potential biomarker (4). In a study of nine esophageal squamous cell carcinoma cell lines, it was found that the methylation level of the *CPNE5* promoter region significantly affected survival and recurrence (5). In 2019, Liang *et al.* found that in five pairs of single-egg twins with discordant Autism Spectrum Disorder (ASD), *SH2B1* had abnormal methylation, which may related to the cause of ASD (6). These studies demonstrate that DNA methylation markers have great application prospects as methods for clinical diagnosis and treatment in the future.

The integration and mining of DNA methylation data generated by high-throughput microarray and sequencing technologies helps researchers discover new candidate disease biomarkers. Current measurement techniques for detecting genomic DNA methylation include approaches to detect gene-specific and genome-wide methylation levels. Gene-specific DNA methylation level quantitative approaches, such as methylation-specific PCR (MSP) (7), bisulfite sequencing PCR(BSP) (8) and MethyLight (9) are widely used in low throughput research. For example, *PTEN* promoter methylation has been found to be significantly associated with age, clinical stage and Her-2 negativity in breast cancer using MSP. Genome-wide DNA methylation detection technologies are varied, and include Illumina Infinium HumanMethylation27 BeadChip (10), Illumina Infinium HumanMethylation450 BeadChip (11), Illumina Infinium HumanMethylation850 BeadChip (12), reduced representation bisulfite sequencing(RRBS) (13), whole-genome bisulfite sequencing(WGBS) (14) and Methylated DNA Immunoprecipitation sequencing(MeDIP-seq) (15). In 2020, the WGBS technique was used in sperm from a Type 2 diabetes mellitus (T2DM) population to identify 10 differentially DNA methylated genes, providing evidence for the first time to explain the complex mechanism of T2DM susceptibility in offspring (16).

In recent years, the increase in data has led to the proliferation of a wide variety of methylation-related databases including: MethBank3.0 (17), MethMotif (18), MethHC2.0 (19) and NGSMethDB (20). In 2018, MethBank3.0 was updated on the basis of 2015 predecessor, integrating consensus reference methylation and single-base resolution methylation. It focuses on humans, other animals, and Plant development, and is equipped with online tools to predict the age of human methylation. In 2019, Benoukraf and colleagues published the MethMotif database (18), from ChIP-seq (21) and WGBS datasets to calculate cell type-specific CpG methylation information and the location of transcription factor binding sites corresponding to mining Inherent connection. NGSMethDB was updated in 2017 to include data on differentially methylated monocytosines and homologous methylated genomic regions of different animals. The database MethHC 2.0 was published in 2021. It focuses on the abnormal methylome of human diseases (especially cancer) and provides RNA expression profiles to support survival analysis based on genes and miRNAs. Furthermore, we developed DiseaseMeth (22) in 2012 and updated it to version 2.0 in 2017 (23). These two databases are the useful source for understanding the molecular mechanisms of human diseases.

To provide users with a better information resource, DiseaseMeth was updated to version 3.0 in this study. It not only focuses on DNA methylation data for all human diseases up to January 2021 but also allows for the mining of characteristics of diseases hidden in the data based on DNA methylation profiles. The DiseaseMeth version 3.0 database contains two types of data: experimental DNA methylation genes with disease associations discovered by manually mining PubMed, and a high-throughput dataset of 162 diseases in the GEO (24) or TCGA (25) database. Furthermore, the tools Methylation Disease Correlation and Cancer Prognosis & Co-Methylation were developed in DiseaseMeth version 3.0 to provide for personalized and comprehensive analyses. In summary, DiseaseMeth version 3.0 intelligently aggregates a large amount of fragmented disease DNA methylation information in public databases and literatures. It also provides a comprehensive analysis database to explore the key role of DNA methylation in human diseases.

### DiseaseMeth version 3.0 design

**Figure 1.**
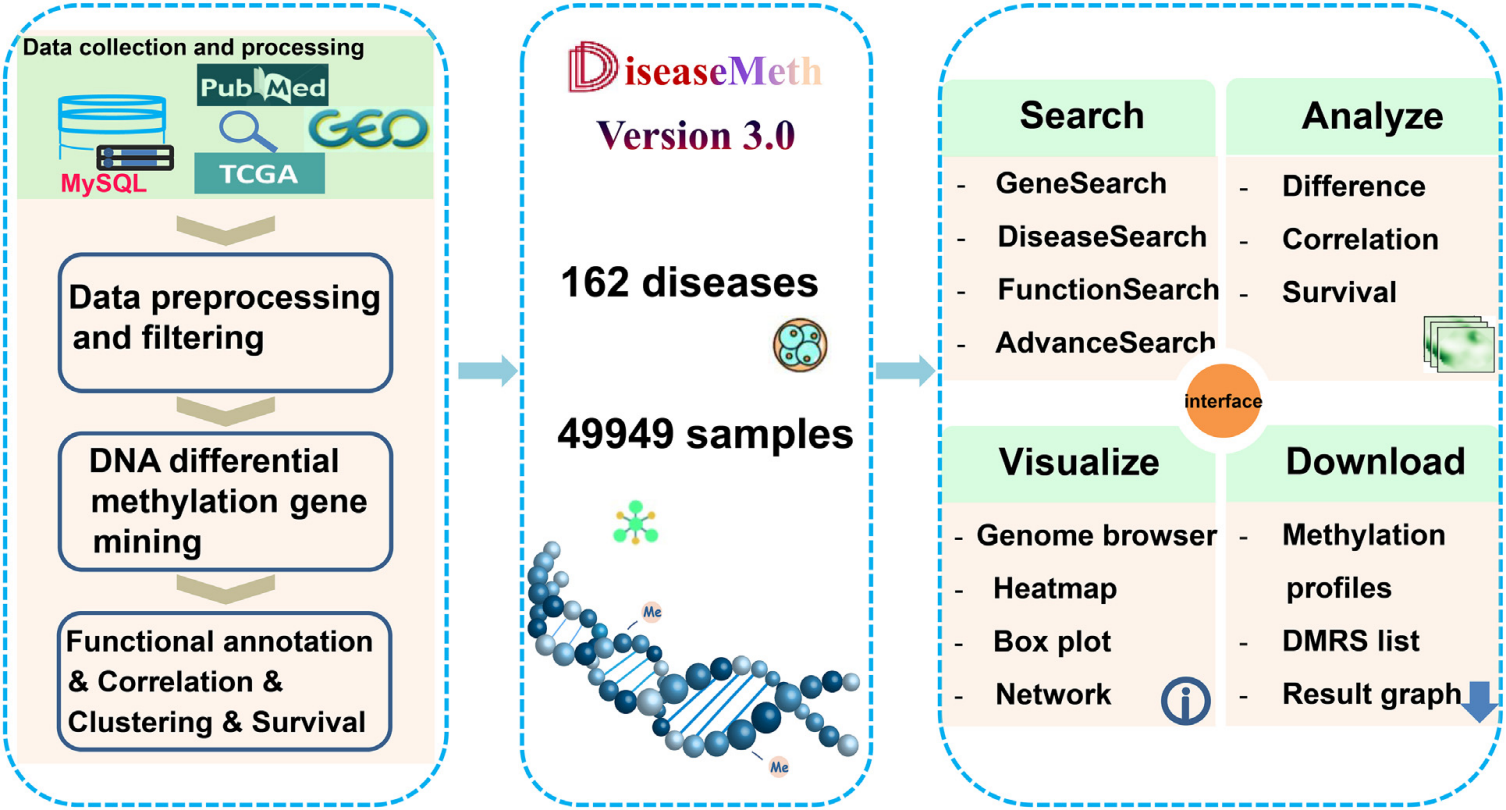
The overall framework of DiseaseMeth3.0 database.

Here, we present DiseaseMeth version 3.0, a comprehensive and curated database that integrates human DNA methylation disease data and metadata from publicly available datasets. Our platform provides the DNA methylation profile of a disease and allows further mining and analyses of that information (Figure 1). It covers a variety of statistical results, including difference analysis, correlation analysis, survival analysis, and network analysis. By optimizing the storage and query methods of its MySQL database, access to DNA methylation information for any disease gene is accelerated. Moreover, a wider, user-friendly interface has been developed to support the main functions of DiseaseMeth version 3.0, including search, interactive exploration, visualization, and download mechanisms. The methylation levels of different genes between disease samples and normal samples in any diseases can also be shown. Specifically for cancer, the methylation level of patients at different stages, as well as patient prognosis and co-methylation module data, can be revealed. Data can be downloaded for free by anyone at the database website.

### Data expansion

DiseaseMeth version 3.0 contains DNA methylation information updated from 1 October 2015 to 31 January 2021, and is based on DiseaseMeth version 2.0. Data sources include public databases and literatures. 4708 samples in 247 related high-throughput datasets were collected from the GEO and TCGA databases. Literature data were searched manually in PubMed resulting in 2210 pairs of associations. In total, 162 disease types and 49 949 samples are recorded in DiseaseMeth version 3.0. Moreover, associations between disease and gene have increased from 679 602 to 1 485 099. The genome-wide dataset contains seven different techniques of DNA methylation detection. The details are shown in Table 1.

**Table 1.** Samples from all DNA methylation sequence platforms in DiseaseMeth version 3.0

			Sample		
Techniques of DNA methylation detection	Dataset	Disease	V 2.0	V 3.0	Total
Whole-Genome Bisulfite Sequencing	16	16	47	209	256
Reduced Representation Bisulfite Sequencing	17	16	35	462	497
Methylated DNA Immunoprecipitation Sequencing	5	5		296	296
Illumina GoldenGate DNA methylation Beadchip	11	12	1265		1265
Illumina Infinium HumanMethylation27 BeadChip	79	44	9016	45	9061
Illumina Infinium HumanMethylation450 BeadChip	252	126	15 948	12790	28 738
Illumina Infinium HumanMethylation850 BeadChip	52	46		3730	3730

### Search section update

Four search approaches are provided in DiseaseMeth version 3.0: GeneSearch, DiseaseSearch, FunctionSearch, and AdvancedSearch. On the GeneSearch page, the gene symbol (gene name/transcript ID) or genome location can be input to obtain the methylation level of that particular gene in the database's disease samples. The output will be displayed as a table and a heatmap. The DNA methylation level of the gene is represented by the heatmap, which can show differences in DNA methylation levels of particular genes in all included diseases. The disease type can be queried on the DiseaseSearch page, and corresponding DNA methylation levels of DMGs for the selected disease are represented in heatmap. Additionally, we developed a new FunctionSearch. Based on GO (26) and KEGG (27), the biological processes and pathways of DMGs are annotated. Functional enrichment information for all DMGs is localized in DiseaseMeth version 3.0. If any diseases of interest are input, functional enrichment results are promptly displayed. Furthermore, additional query parameters including gene symbol, GO term, and pathway ID, can be used as more precise query requirements. The AdvanceSearch page allows for even more specific queries. One or more qualified entry, that is, gene name/transcript ID, genome location, disease type, and technology, can be input to help users obtain desired datasets rapidly.

### Standard pipeline to determine and analyze differential DNA methylation genes

**Figure 2.**
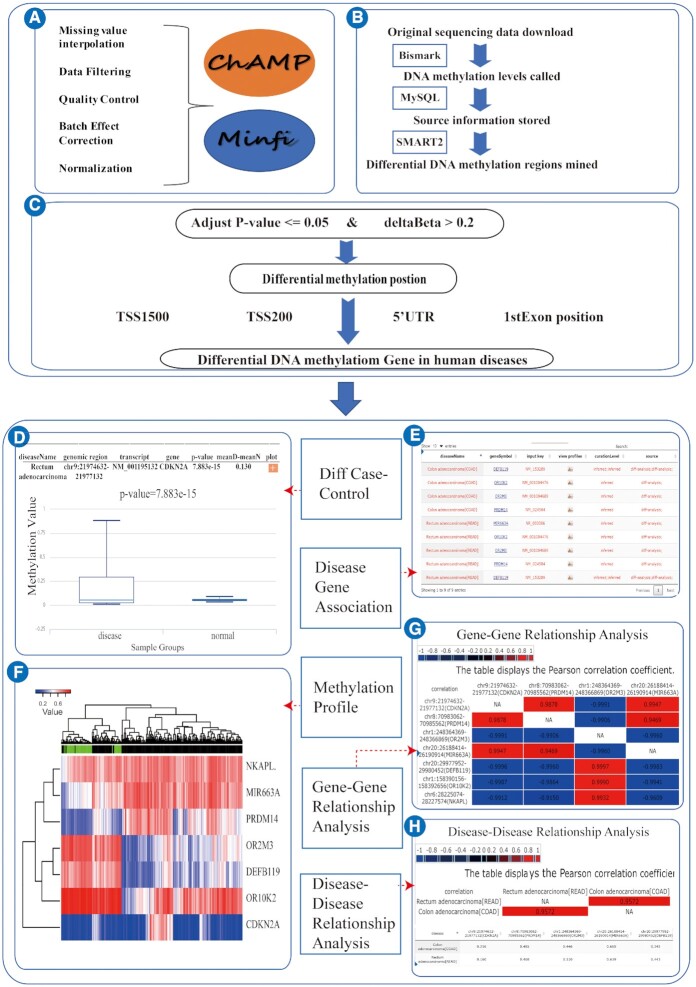
The pipeline to identify specific differential DNA methylation gene. (**A**) Algorithm to determine differential DNA Methylation genes from array sequencing platform. (**B**) Algorithm to determine differential DNA Methylation genes from whole-genome sequencing platform. (**C**) The threshold of determining differential DNA methylation regions. (**D**) Differential analysis between case and control. (**E**) Association between disease and methylation of gene. (**F**) Methylation profile. (**G**) Gene–gene relationship analysis. (**H**) Disease–disease relationship analysis.

For ease of use and to reveal more information, we have built a unified process, standardized pipeline to analyze DNA methylation data. Using combined Illumina Infinium HumanMethylation27 BeadChip, Illumina Infinium HumanMethylation450 BeadChip, and Illumina Infinium HumanMethylation850 BeadChip data from array techniques, we analyzed the data using the following steps (Figure 2A):

- Downloaded the raw data and reference platform data from public databases.
- Uniformly represented the DNA methylation level as a β value.
- Integrated DNA methylation data from different batches or different databases with the R package ‘sva’ (28) to eliminate batch effects.
- Used the KNN algorithm (29) to fill in missing values to ensure that high-quality probes with rich methylation levels are used to ensure more accurate results through differential analyses.
- Used the R package ‘ChAMP’ (30) and ‘minfi’ (31) to analyze and identify DNA methylation differential sites and regions. Our standard for identification is that the difference in the β value mean between two sets of samples is greater than 0.2, and the corrected *P*-value is <0.05.
- Retained the intersection of differentially methylated sites located in promoter regions (TSS1500, 5′UTR, 1stExon, and TSS200)(Figure 2C).
- Defined the average value of probe methylation levels of all the promoter regions corresponding to each gene as its methylation level.

For DNA methylation sequencing data, including WGBS and RRBS, we analyzed the data using the following steps (Figure 2B):

- Downloaded the original sequencing data of the corresponding subsequence platform.
- Used Bismark (32) to map bisulfite treated sequencing reads to Genome Reference Consortium Human Build 38 (GRCh38) and
- extract the methylation value.
- iii) Used SMART2 (33) to mine differential DNA methylation regions between disease and control samples.
- iv) Retained the intersection of differentially methylated regions located in promoter regions (TSS1500, 5′UTR, 1stExon and TSS200).
- v) Calculated the DNA methylation level of genes with the average value of differentially methylated regions.

An analyze section was developed in DiseaseMeth version 3.0 to deepen the exploration of genes obtained through the above pipeline. In this section, differences in DNA methylation levels of a query gene in various diseases can be displayed intuitively in the form of a table, and the differences in DNA methylation levels between two sets of samples can be visualized in the form of box plots (Figure 2D) and heatmaps (Figure 2F). Primary results in the result table include probe ID, significance *P* value, and corrected *P* value, along with the very important result, that of which gene symbol corresponds to the probe. Chromosome location information for the probe is located above these results (Figure 2E). When selecting multiple diseases in Analyze section, users can enter a list of gene symbols to obtain the DNA methylation correlations of the common differential genes between the diseases (Figure 2G), as well as correlations between diseases, expressed in the form of tables and correlation heatmaps (Figure 2H). In total we identified 22 718 genes in 99 diseases with significant differential DNA methylation from all of our collected array and high-throughput sequencing data.

### Tools development and update

Because DNA methylation can provide biomarkers to refine the human disease blueprint, DiseaseMeth version 3.0 provides two independently developed tools: Methylation Disease Correlation and Cancer Prognosis & Co-Methylation.

**Figure 3.**
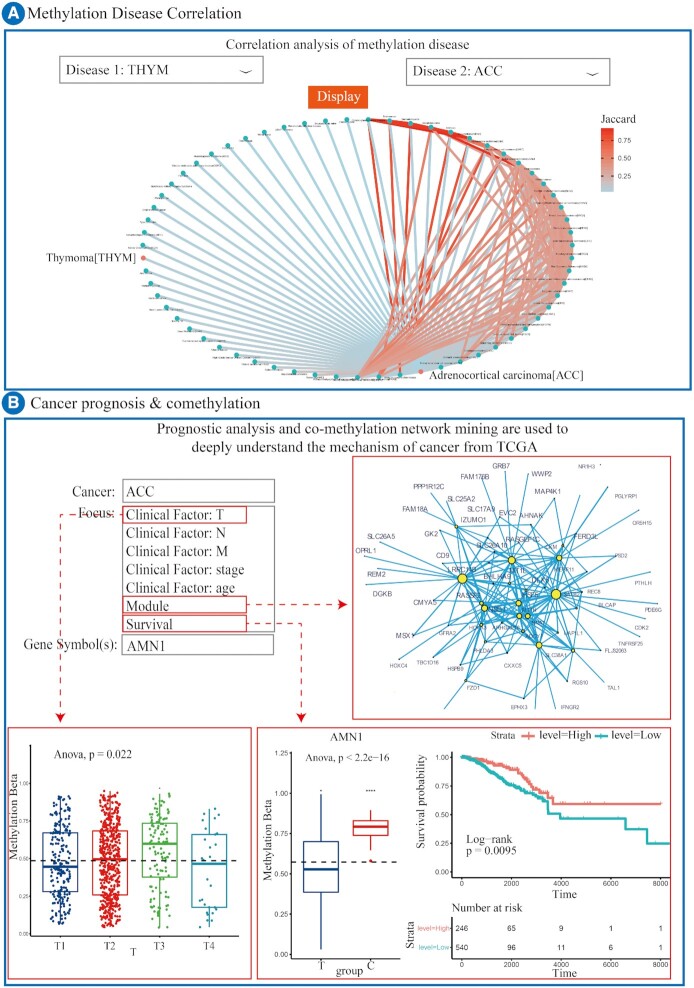
Overview of tools in DiseaseMeth version 3.0. (**A**) Correlation analysis of methylation diseases. (**B**) Cancer prognosis & co-methylation.

As research deepens, diseases in different tissues might show similar global DNA methylation patterns (34,35). Therefore, we developed a network analysis tool across diseases, Methylation Disease Correlation, to explore the correlation between diseases mediated by DNA methylation (Figure 3A). This tool allows a user to obtain the correlation across diseases by calculated Jaccard similarity test using the intersections and mergers of DMGs pairwise for all 99 diseases with 22 718 DMGs. If there is a significant association between two diseases (*P* < 0.05, Jaccard > mean ± sd), then those two diseases are connected to form a disease association network. For diseases in the network that are linked in one step to a disease, the Jaccard coefficients of any two of these diseases can be screened (Jaccard > 0.6) to form the complete whole disease association network mediated by DNA methylation (WDAN).

Additionally, perturbations of DNA methylation patterns, such as the methylation of CGI promoters for tumor suppressor genes, are frequently observed in cancer (36). These perturbations imply a cancerogenic role. DiseaseMeth version 3.0 provides the Cancer Prognosis & Co-Methylation tool to mine key DNA methylation genes in 31 types of cancer from the TCGA database by both survival analysis and gene module mining. A dropdown menu can be used in the Cancer Prognosis & Co-Methylation tool (Figure 3B) to select a cancer name, clinical factor T, clinical factor N, clinical factor M, clinical factor stage, clinical factor age, survival, or module. This tool illustrates differences in DNA methylation levels between different types of genes/multi-genes using box plots to provide clinical characteristics. For example, one could select clinical factor T to see the differences in DNA methylation levels of the input gene among T1, T2, T3 and T4. We also provide a newly self-developed online survival analysis tool. Log-rank test and Kaplan–Meier curves (37) reflect the difference in survival between patients with different DNA methylation levels for a particular input gene. Furthermore, co-methylation modules can be mined from the DMGs co-methylation network. To illustrate this, we performed Pearson correlation analysis across the DMGs in all of the cancers in our database, retaining gene pairs with *P* < 0.01, cor > 0.6 to form a co-methylation network of 31 cancers, respectively. The modules were mined using the R package ‘igraph’ (38). Due to limitations of picture clarity using a network module with an excess of genes, only the top 250 relationship pairs with the largest correlation coefficient are displayed. A text version file of the module list can also be downloaded.

### Discussion and future development

We have been committed to discovering key DNA methylation markers in disease and providing reliable support for extensive biomedical research in the field since the establishment of the DiseaseMeth database in 2012. Therefore, we proposed to upgrade DiseaseMeth with substantial innovations into version 3.0. A primary factor in our decision was that the amount of methylation data had increased tremendously since version 2.0. At the time of writing, the number of disease types has increased from 88 to 162. The total number of samples has increased from 32,701 to 49,949, all with high-throughput profiles. Additionally, we added FunctionSearch to the search section. It directly and quickly provides functional annotation information of selected diseases and genes from the localized GO or KEGG enrichment. Importantly, we conducted a detailed analysis of collected cancer patient clinical data to explore the impact of DNA methylation on patient prognosis, and to demonstrate the methylation levels of different pathological and clinical features of tumors. Fast and stable visualizations are available in every section of the database, whether it is a box plot, a heatmap, or a correlation network diagram. Finally, a new independent R package survival tool was developed in DiseaseMeth version 3.0. This new tool overcomes the limitations of personalized analyses, unlike the survival analysis tool in DiseaseMeth version 2.0 that was based on PROGgene (39).

The cost of DNA methylation microarrays and high-throughput sequencing continues to decline; methylation sequencing may gradually become mainstream. The increase in MeDIP-seq data as been relatively slow since 2015; therefore, we did not consider it when developing our standard pipeline to determine and analyze differential DNA Methylation genes. For DNA methylation data processing and analysis by MeDIP-seq technology can be used Model-based Analysis of Chip-Seq (MACS) (40) to call peaks related to gene and quantitative differentially methylated regions (QDMR) (41) to identify the DMGs. Because not all 162 diseases have DMGs that intersect, and the experimental data don’t have methylation level, these were not used in the Methylation Disease Correlation tool. As our dataset increases in size with future development, the resulting disease networks will enable inquiry of more and more methylation associations across diseases.

In the future, as more researchers focus on DNA methylation, more data will be presented and more in-depth research on DNA methylation mechanisms and regulation in disease will occur. Molecular features associated with DNA methylation such as expression, chromatin structure variation, and mutation are also research topics of concern. We will continue to refine and add more information to our platform and further reveal the impact of epigenetics on disease. DiseaseMeth version 3.0 not only provides a data source for studying the epigenetic regulation of disease mediated by DNA methylation, but also comprehensively characterizes DNA methylation as a biomarker of disease.

## DATA AVAILABILITY

DiseaseMeth version 3.0 is an open source database, which is freely available at http://diseasemeth.edbc.org.
